# Effect of Neonatal Treatment With the NMDA Receptor Antagonist, MK-801, During Different Temporal Windows of Postnatal Period in Adult Prefrontal Cortical and Hippocampal Function

**DOI:** 10.3389/fnbeh.2021.689193

**Published:** 2021-06-11

**Authors:** Maria E. Plataki, Konstantinos Diskos, Christos Sougklakos, Marouso Velissariou, Alexandros Georgilis, Vasiliki Stavroulaki, Kyriaki Sidiropoulou

**Affiliations:** ^1^Department of Biology, University of Crete, Heraklion, Greece; ^2^Institute of Molecular Biology and Biotechnology—Foundation for Research and Technology Hellas, Heraklion, Greece

**Keywords:** object recognition, social behavior, contextual fear conditioning, up states, neuronal oscillations, parvalbumin interneurons

## Abstract

The neonatal MK-801 model of schizophrenia has been developed based on the neurodevelopmental and NMDA receptor hypofunction hypotheses of schizophrenia. This animal model is generated with the use of the NMDA receptor antagonist, MK-801, during different temporal windows of postnatal life of rodents leading to behavioral defects in adulthood. However, no studies have examined the role of specific postnatal time periods in the neonatal MK-801 (nMK-801) rodent model and the resulting behavioral and neurobiological effects. Thus, the goal of this study is to systematically investigate the role of NMDA hypofunction, during specific temporal windows in postnatal life on different cognitive and social behavioral paradigms, as well as various neurobiological effects during adulthood. Both female and male mice were injected intraperitoneally (i.p.) with MK-801 during postnatal days 7–14 (p7–14) or 11–15 (p11–15). Control mice were injected with saline during the respective time period. In adulthood, mice were tested in various cognitive and social behavioral tasks. Mice nMK-801-treated on p7–14 show impaired performance in the novel object, object-to-place, and temporal order object recognition (TOR) tasks, the sociability test, and contextual fear extinction. Mice nMK-801-treated on p11–15 only affects performance in the TOR task, the social memory test, and contextual fear extinction. No differences were identified in the expression of NMDA receptor subunits, the synapsin or PSD-95 proteins, either in the prefrontal cortex (PFC) or the hippocampus (HPC), brain regions significantly affected in schizophrenia. The number of parvalbumin (PV)-expressing cells is significantly reduced in the PFC, but not in the HPC, of nMK-801-treated mice on p7–14 compared to their controls. No differences in PV-expressing cells (PFC or HPC) were identified in nMK-801-treated mice on p11–15. We further examined PFC function by recording spontaneous activity in a solution that allows up state generation. We find that the frequency of up states is significantly reduced in both nMK-801-treated mice on p7–14 and p11–15 compared to saline-treated mice. Furthermore, we find adaptations in the gamma and high gamma activity in nMK-801-treated mice. In conclusion, our results show that MK-801 treatment during specific postnatal temporal windows has differential effects on cognitive and social behaviors, as well as on underlying neurobiological substrates.

## Introduction

Schizophrenia (SZ) is a neuropsychiatric disorder affecting 1% of the world population [data from World Health Organization (WHO), 2019]. SZ symptoms can be positive (hallucinations, delusions, and thought disorders), negative (reduced reward sensitivity and social interaction defects; Sawa and Seidman, 2014), and cognitive (deficits in attention and in working or long-term memory; Davidson et al., 1999). Besides significant developments on the genetic basis of SZ (Birnbaum and Weinberger, 2017), the diagnosis is still based only on clinical features. Furthermore, pharmacological interventions have a very limited effect on cognitive functions, in particular. Brain regions that are significantly affected in SZ patients and in animal models of SZ include the prefrontal cortex (PFC) and the hippocampus (HPC), both of which are important for cognitive function and control (Wang et al., 2009).

There are three main hypotheses for the etiology of SZ: (a) the dopamine hypothesis (excessive dopaminergic transmission; Davis et al., 1991; Howes and Kapur, 2009; Brisch et al., 2014); (b) the glutamate hypothesis (hypofunction of NMDA receptors; Olney et al., 1999; Snyder and Gao, 2013; Nakazawa and Sapkota, 2020); and (c) the neurodevelopmental hypothesis (disturbing the central nervous system development; Lafargue and Brasic, 2000; Marenco and Weinberger, 2000; Fatemi and Folsom, 2009; Owen et al., 2011). Currently, the leading hypothesis is neurodevelopmental that postulates defective development of neuronal populations. Important developmental processes regarding brain maturation, such as synapse formation, axon elongation, and maturation of neurotransmission, begin during the gestational period, but continue during the early postnatal days in both rodents and humans (Kolb and Whishaw, 1989; Viberg, 2009; Teffer and Semendeferi, 2012). Given the fact that NMDA receptor blockade can lead to SZ-like behaviors in both humans and rodents, it is hypothesized that NMDA receptor blockade, during a specific postnatal period, could result in SZ-like behaviors during adulthood. It has been shown that MK-801 treatment on postnatal day 7 leads to an increase of neuronal apoptosis on postnatal day 8 (Ikonomidou et al., 1999; Coleman et al., 2009), which does not continue upon removal of the NMDA receptor blocker (Ringler et al., 2008).

Several studies have administered the NMDA receptor antagonist, MK-801, in rodents during early postnatal life and have subsequently tested rodent behavior in adulthood. However, the studies are inconsistent with regards to the postnatal duration for treatment, frequency, and dose of MK-801.

Neonatal treatment with MK-801 during the temporal windows of p1–22 (Facchinetti et al., 1993), p3 (Beninger et al., 2002), p7 (Harris et al., 2003), p6–21 (Schiffelholz et al., 2004), p5–14 (Guo et al., 2010), p11 (Fredriksson and Archer, 2004) led to long-term changes in locomotor activity when compared to controls. Relatively long-term, but starting after the first postnatal week, neonatal administration of MK-801 (p7–20) resulted in deficits in spatial learning and memory in adulthood (McLamb et al., 1990; Gorter and de Bruin, 1992; Kawabe et al., 2007). On the other hand, more time-limited treatment with MK-801, during p7–10 did not have an effect on locomotor activity or novel object recognition, but resulted in impaired cognitive flexibility and working memory (Stefani and Moghaddam, 2005).

Similar MK-801 treatments, such as p7–10 (Uehara et al., 2009) and p7 (Harris et al., 2003) led to deficits in prepulse-inhibition (PPI), a neurophysiological index of sensorimotor gating function, whose deficits are typically present in people with SZ. However, another study with similar MK-801 treatment on p6–8–10 (Lyall et al., 2009) had no long-term effect on PPI.

At the molecular level, p7 or p6–21 MK-801-treated rats were characterized by altered levels of NR1 in the HPC and other cortical regions (Harris et al., 2003; Baier et al., 2009), while MK-801 treatment at p7 (Coleman et al., 2009) caused a reduction of PV positive GABAergic interneurons in PFC of adult mice. On the other hand, MK-801 treatment during p1–22 (Facchinetti et al., 1993) did not alter glutamate decarboxylase during adulthood.

Given the great temporal variability in MK-801 treatment, our goal in this study is to systematically investigate the long-term effects of neonatal administration of MK-801, during different and specific developmental windows. We investigated several behavioral paradigms, including different types of object recognition tasks, sociability, and contextual fear conditioning, and extinction. Furthermore, we studied the expression levels of NMDA receptor subunits and synaptic proteins, the number of PV-expressing cells in the PFC and HPC, and the properties of spontaneous activity in the PFC.

## Materials and Methods

### Animals

All experiments were conducted in adult (>3 months old) C57BL/6 female and male mice. Mice were bred in the animal facility of the Department of Biology, University of Crete, housed in same-sex groups (3–4 per cage) and provided with standard mouse chow and water *ad libitum*, under a 12 h light/dark cycle (light on at 7:00 a.m.) with controlled temperature (23 ± 1°C). All procedures were performed according to protocols approved by the Research Ethics Committee of the University of Crete and follow the European Union ethical standards outlined in the Council Directive 2010/63/EU of the European Parliament on the protection of animals used for scientific purposes.

For the neonatal MK-801 mouse models (nMK-801), both female and male C57BL/6 mice were injected intraperitoneally (i.p.) once daily with 0.1 mg/kg of MK-801, during postnatal days 7–14 (p7–14) or 11–15 (p11–15). Control groups were injected i.p. with the same volume of saline at the respective time period. Mice were allowed to reach adulthood (after p90), before the initiation of the experiments.

### Behavioral Tasks

**Figure 1:**
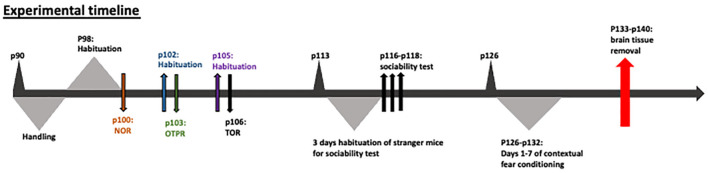
Experimental design.

All mice underwent the following behavioral tasks in the following order: (1) Novel object recognition; (2) Object-to-place recognition; (3) Temporal order object recognition (TOR); (4) Sociability test; and (5) Contextual fear conditioning and extinction. The order of the experiments was selected on the basis that the least intense tasks be conducted prior to the most intense tasks, such as fear conditioning, which was the last task. The sequence of the tasks did not affect the behavior as indicated by the animals’ performance, in comparison with our average animal performance in our facility. Before behavioral testing was initiated, each mouse was familiarized with the experimenter for 7 days before the behavioral experiments. The experimental design can be visualized in Figure 1.

### Novel Object Recognition Task

On p90 each mouse was habituated for 15 min in an open field 45 × 45 × 45 cm, once a day for 3 days. On p100, two identical objects were placed into the open field and the mouse was left to explore them for 5 min. After 25 min, the mouse was placed back in the open field chamber for 5 min in order to explore one familiar object from the previous trial and one new object. The time of object exploration was measured using JWatcher. Object exploration was defined as the time spent exploring the objects by physical proximity (i.e., touching, sniffing). The object exploration index was calculated as: [(novel object exploration time + familiar object exploration time)/total time in the chamber]. The discrimination index of the test phase was calculated as: [(time exploring the novel object − time exploring the familiar object)/(time exploring the novel object + time exploring the familiar object)].

### Object to Place Recognition Task

On p102, a 15-min habituation took place. The following day (p103), each mouse was left to explore for 5 min two new identical objects in the open field. After a 25-min break, one of the previously introduced objects was placed at a new position (displaced object), while the other one remained at the same position (stationary) within the open field, and the mouse was left to explore the open field for 5 min. The object exploration index and the discrimination index of the test phase were measured as previously described.

### Temporal Order Object Recognition (TOR) Task

On p105 one more habituation took place, while on p106 the animals were subjected to two sample trials and one test trial with an inter-trial interval of 25 min. In each sample trial, the animals explored two identical objects for 5 min, a different pair of identical objects for each trial. During the test trial, one object from sample trial 1 (less familiar) and one object from sample trial 2 (familiar) were presented, and the animals were allowed to explore the open field for 5 min. The trial and test phases were recorded and analyzed offline. The time that mice explored the two different objects was measured and the discrimination and exploration indices were calculated as described above.

### Sociability Test

On p116 or p117 or p118, the sociability test took place in a 60 × 40 × 22 cm (height) device, which consists of three communicating compartments, 20 × 40 × 22 each one. Small, door-like openings allowed for free access to each chamber. “Stranger” mice (C57BL/6; totally newly introduced mice to the experimental mice) were habituated for 10 min into a cylindrical box with openings all around, every day for 3 days before the experiment. The sociability test was comprised of four successive phases. Firstly, the experimental mouse was placed into the middle compartment and allowed to habituate for 10 min, while the doors leading to the other compartments were closed. During phase I the mouse was habituated into the device with free access to all three compartments for 10 min. During phase II, one stranger mouse of the same sex as the experimental mouse was placed into the cylinder box in one side compartment and an empty box was placed into the other side compartment. The experimental mouse was left to explore the whole device for 10 min. The time of exploration was measured using JWatcher, while the discrimination index for each mouse was calculated as [(time spent exploring the stranger mouse − time spent exploring the empty box)/(time spent exploring the stranger mouse + time spent exploring the empty box)].

During the social memory test (phase III of sociability test), apart from the first stranger mouse (familiar stranger mouse from phase II), a new stranger mouse was placed inside the previously empty cylindrical box. The new stranger mouse was similarly of the same sex as the experimental one and the experimental mouse was let to explore the device. The time of exploration was measured using JWatcher, while the discrimination index for each mouse was calculated as [(time exploring the newly introduced stranger mouse − time exploring the familiar stranger mouse)/(time exploring the new stranger mouse + time exploring the familiar stranger mouse)].

### Contextual Fear Conditioning/Fear Memory Extinction

On p126, the contextual fear conditioning experiment was conducted. During the training day of the contextual fear conditioning, the mouse was placed in the fear conditioning chamber. On the 7th minute, the mouse received an electrical shock of 0.75 mA for 1 s through the metal floor bars. On days 1–6 after the training day, the mouse was placed back in the chamber for 10 min every day. For the fear conditioning, the time the mouse spent freezing was measured by JWatcher during the 1–3 min of days 1–6. The percentage of freezing was calculated as: [(times found not moving*100)/(times found moving + times found not moving)].

On p133–140 the mice brains were removed for one of the following 3 procedures: (a) Western blotting analysis; (b) parvalbumin immunofluorescence; and (3) acute brain slice electrophysiology.

### Western Blot

Mice were sacrificed after anesthesia with halothane for PFC and HPC extraction in dry ice. For protein extraction, Lysis buffer was used (50 mM HEPES/150 mMNaCl/1%Glycerol/1%Triton-X-100/1.5 mM MgCl_2_/5 mM EGTA/1:1,000 protein inhibitors) and the mixture was sonicated for 10 s (Amplitude 40). After a high-speed centrifuge for 30 min, the supernatant was isolated and stored at −80°C. Forty microgram of the protein were diluted 1:1 with 2× sample buffer (0.125 M Tris pH = 6.8/4% SDS/0.04 M DTT/10% Glycerol/3 μM Bromophenol blue) and ran in a 10% Acrylamide gel. Then, proteins were transferred to a nitrocellulose membrane and blocked with a blocking buffer (5%BSA in 0.1% TBST). Following, membranes were incubated in primary antibody (in 5%BSA/0.1% TBST) O/N at 4°C [NR1(Cell Signaling) 1:500, NR2A/B (Cell Signaling) 1:1,000, PSD95 (Cell Signaling) 1:1,000, SYNAPSIN (Cell Signaling) 1:1,000, GAPDH (Cell Signaling) 1:2,000]. The next day after washes, membranes were incubated in a secondary antibody [goat anti-rabbit (Invitrogen) 1:5,000] and visualized using Sapphire Biomolecular Imager of Azure biosystems. The protein band volume was measured using ImageLab 6.1 program and then normalized.

### Immunofluorescence

Mice were transcardially perfused and the brain was fixed with 4% PFA. Then, 40 μm slices were incubated in blocking buffer with 10% FBS/0.4% Triton/0.1% TBST and then used for PV detection in PFC and HPC intersections by incubating them in anti-PV antibody (rabbit-polyclonal anti-PV, 1:3,000, PV27, Swant, Inc., Switzerland; 1:3,000) O/N in 2% FBS/0.4% Triton/0.1% TBST. A fluorescent secondary antibody (Goat-anti-rabbit, Alexa488 conjugated, 1:500, Thermo Fisher Scientific, Inc., Waltham, MA, USA) was used for the detection of PV expression using an inverted confocal microscope. Finally, the slices were incubated with RNAase (Qiagen; 1:200) and then with propidium iodide (ThermoFisher Scientific). The number of cells was calculated by measuring the number of cells in PFC (anterior cingulate and prelimbic areas) and HPC (CA1 region).

### Electrophysiology

Mice were euthanized under halothane anesthesia, the brain was removed and placed in ice-cold oxygenated artificial cerebrospinal fluid (aCSF; 95% O_2_/5% CO_2_) containing (in mM): 125 NaCl, 3.5 KCl, 26 NaHCO_3_, 1 MgCl_2,_ and 10 glucose (pH = 7.4, 315 mOsm/l). The brain was blocked and the part containing the PFC was placed onto the stage of a vibratome (Leica, VT1000S, Leica Biosystems GmbH, Wetzlar, Germany). The brain slices (400 μm) containing the PFC (Bregma 2.22–1.70 mm) were placed in a submerged chamber containing oxygenated (95% O_2_/5% CO_2_) aCSF (in mM): 125 NaCl, 3.5 KCl, 26 NaHCO_3_, 2 CaCl_2_, 1 MgCl_2,_ and 10 glucose (pH = 7.4, 315 mOsm/l) at 37°C. Brain slices containing the PFC were taken using a vibratome in ice-cold oxygenated aCSF and then incubated for at least 45 min in a modified aCSF that allows the generation of up states in brain slices (aCSF containing, in mM: 125 NaCl, 4.5 KCl, 26 NaHCO_3_, 1 MgCl_2_, 1 CaCl_2_, and 10 glucose (pH = 7.4, 315 mOsm/l) at 37°C with 95% O2–5% CO_2_ (Sanchez-Vives and McCormick, 2000).

The recording electrode was filled with 3M NaCl and was located in layer II of the PFC. Spontaneous field potentials were amplified using the EXT-02F amplifier (National Instruments), digitized with ITC-18 (Instrutech, Inc.), and recorded in a computer running Windows10 with WinWCP software (Stratchclyde electrophysiology software). Extracellular recordings were performed for at least 10 min for each condition per brain slice, firstly without a high-pass filter, and immediately afterward with a 3 Hz high-pass filter. The 3 Hz high-pass filter recordings were used to study the frequency of up states, while the no high-pass filter recordings were used to study neuronal oscillations.

Neuronal oscillations were analyzed as reported previously (Kalemaki et al., 2018). Twenty traces of spontaneous voltage signals (32 s each trace) were used from each brain slice. The voltage signals were decomposed using discrete Fourier transformations. The power spectra of different frequency domains (Delta: 1–4 Hz, Theta: 4–7 Hz, Alpha: 8–12 Hz, Beta: 13–30 Hz, Gamma: 30–80 Hz, High Gamma: 80–150 Hz) were generated using custom-written procedures in MATLAB-R2020a (The MathWorks, Inc.). The rate of power spectrum that exists in each frequency is computed by the equation:

$$\text{Rate of Power (\%) = sum}\frac{\text{Power (specific oscillation)}}{\text{total (Power)}}\%$$

which is the signal rate of each oscillation in relation to the whole signal and shows for each oscillation the ratio of energy that exists in the specific range.

### Statistics

We first examined the normality of the data using the Kolmogorov-Smirnov’s test. Two-way ANOVA analysis was used in the data from the behavioral analysis, with the two-factors being sex and treatment. Repeated-measures ANOVA was used to analyze the data from contextual fear conditioning. Data from wWstern blots and immunofluorescence were analyzed using the *t*-test, while data from electrophysiology were analyzed with one-way ANOVA. When the one-way ANOVA was significant or the interaction of two-way ANOVA was significant, the LSD *post hoc* test was used.

## Results

### Object Recognition Tasks

**Figure 2:**
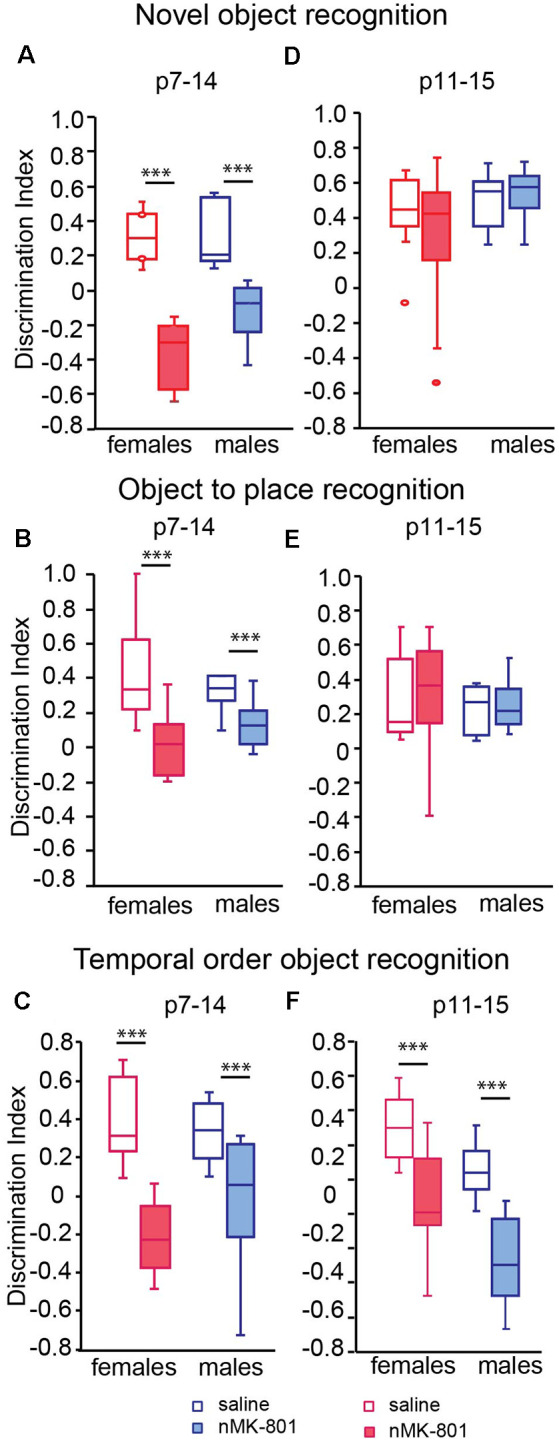
Effects of neonatal treatment with MK-801 at p7–14 (*n* = 15) and p11–15 (*n* = 15) on the novel object, object to place, and temporal order object recognition (TOR; *n* = 13 for each saline-treated group). Graphs showing the discrimination index in the novel object recognition **(A,D)**, the object to place **(B,E)**, and the TOR **(C,F)** for adult mice neonatally treated with MK-801 during p7–14 **(A–C)** and p11–15 **(D–F)**. Open bars represent saline-treated mice, while colored bars represent the nMK-801-treated mice. **(A)** In the novel object recognition test, there was a significant effect of nMK-801 (p7–14) treatment (*F*_(1,26)_ = 77.621, *p* < 0.001), but not effect of sex (*F*_(1,26)_ = 3.466, *p* = 0.075) or sex*treatment interaction (*F*_(1,26)_ = 2.282, *p* = 0.144). **(B)** In the object-to-place recognition test, there a significant effect of nMK-801 (p7–14) treatment (*F*_(1,26)_ = 16.856, *p* < 0.001), but not an effect of sex (*F*_(1,23)_ = 0.002, *p* = 0.962) or sex*treatment interaction (*F*_(1,26)_ = 2.201, *p* = 0.150). **(C)** In the TOR test, there was a significant effect of nMK-801 (p7–14) treatment (*F*_(1,26)_ = 26.124, *p* < 0.001), but not an effect of sex (*F*_(1,23)_ = 0.544, *p* = 0.467) or sex*treatment interaction (*F*_(1,26)_ = 2.259, *p* = 0.129). **(D)** In the novel object recognition test, there was no effect of sex (*F*_(1,26)_ = 1.317, *p* = 0.263), nMK-801 (p11–15) treatment (*F*_(1,26)_ = 0.170, *p* = 0.682) or sex*treatment interaction (*F*_(1,26)_ = 0.733, *p* = 0.397).** (E)** In the object-to-place recognition test, there was no effect of sex (*F*_(1,26)_ = 0.380, *p* = 0.543), treatment nMK-801(p11–15; *F*_(1,26)_ = 0.022, *p* = 0.883) or sex*treatment interaction (*F*_(1,26)_ = 0.035, *p* = 0.853). **(F)** In the TOR, there was a significant effect of nMK-801(p11–15) treatment (*F*_(1,26)_ = 34.378, *p* < 0.001), of sex (*F*_(1,26)_ = 7.781, *p* = 0.002) but not a significant effect of sex*treatment interaction (*F*_(1,26)_ = 0.429, *p* = 0.512). ***p < 0.001.

Both female and male saline (p7–14)-treated mice explore mostly the novel object during the novel object recognition task. However, both female and male of nMK-801 (p7–14)-treated mice were unable to discriminate between the new and the familiar object, resulting in a significant reduction of discrimination index [Figure 2A; significant effect of nMK-801(p7–14) treatment (*F*_(1,26)_ = 77.621, *p* < 0.001), but not effect of sex (*F*_(1,26)_ = 3.466, *p* = 0.075) or sex*treatment interaction (*F*_(1,26)_ = 2.282, *p* = 0.144)]. In the object-to-place recognition task, female and male saline (p7–14)-treated mice spent more time exploring the displaced than the stationary object, compared to nMK-801(p7–14)-treated mice, both female and male, which exhibited a significant decrease in the discrimination index [Figure 2B; significant effect of nMK-801(p7–14) treatment (*F*_(1,26)_ = 16.856, *p* < 0.001), but not an effect of sex (*F*_(1,23)_ = 0.002, *p* = 0.962) or sex*treatment interaction (*F*_(1,23)_ = 2.201, *p* = 0.150)]. In the TOR task, saline (p7–14)-treated mice preferred to explore the less familiar object (from sample trial 1), while nMK-801 (p7–14)-treated mice did not show any preference between the two objects. As a result, nMK-801 (p7–14)-treated mice exhibit significantly reduced discrimination index [Figure 2C; significant effect of nMK-801 (p7–14) treatment (*F*_(1,26)_ = 26.124, *p* < 0.001), but not an effect of sex (*F*_(1,23)_ = 0.544, *p* = 0.467) or sex*treatment interaction (*F*_(1,23)_ = 2.259, *p* = 0.129)].

In the novel object recognition task, saline (p11–15)-treated mice explored mostly the new object compared to the familiar one, as expected. The same performance was observed in nMK-801 (p11–15)-treated mice, for either female or male mice. Thus, no significant differences in discrimination index between saline (p11–15)-treated and nMK-801 (p11–15)-treated mice were identified [Figure 2D; no effect of sex (*F*_(1,26)_ = 1.317, *p* = 0.263), nMK-801 (p11–15) treatment (*F*_(1,26)_ = 0.170, *p* = 0.682) or sex*treatment interaction (*F*_(1,26)_ = 0.733, *p* = 0.397)].

In object-to-place recognition task, both saline (p11–15)-treated and nMK-801 (p11–15)-treated mice (both female and male) spent more time exploring the displaced object compared to the stationary one. Thus, no significant differences regarding the discrimination indices among the different groups were identified [Figure 2E; no effect of sex (*F*_(1,26)_ = 0.380, *p* = 0.543), treatment nMK-801 (p11–15; *F*_(1,26)_ = 0.022, *p* = 0.883) or sex*treatment interaction (*F*_(1,26)_ = 0.035, *p* = 0.853)]. In the TOR task, saline (p11–15)-treated mice interacted mostly with the less familiar object (from sample trial 1) compared to the familiar object (from sample trial 2), as expected. Both female and male nMK-801 (p11–15)-treated mice were characterized by significantly reduced discrimination indices compared to saline (p11–15)-treated mice [Figure 2F; significant effect of nMK-801 (p11–15) treatment (*F*_(1,26)_ = 34.378, *p* < 0.001), of sex (*F*_(1,23)_ = 7.781, *p* = 0.002) but not a significant effect of sex*treatment interaction (*F*_(1,26)_ = 0.429, *p* = 0.512)].

### Social Behavior Tests

**Figure 3:**
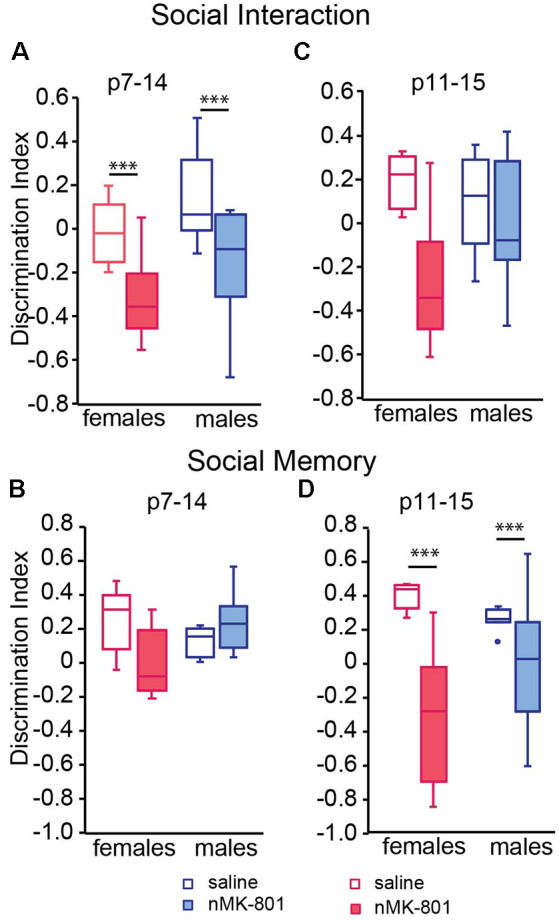
Effects of neonatal treatment with MK-801 at p7–14 **(A,B)** (*n* = 15) and p11–15 **(C,D)** (*n* = 15) compared to the respective saline-treated mice (*n* = 13 for each saline-treated group) on sociability and social memory. Open bars represent saline-treated mice, while colored bars represent the nMK-801-treated mice. **(A)** In the sociability test, there was a significant effect of nMK-801 (p7–14) treatment (*F*_(1,26)_ = 15.264, *p* = 0.001), but not effect of sex (*F*_(1,26)_ = 3.809, *p* = 0.061) or sex*treatment interaction (*F*_(1,26)_ = 0.013, *p* = 0.909). **(B)** In the social memory test, there was a significant effect of sex*treatment interaction (*F*_(1,26)_ = 10.303, *p* = 0.003), but no effect of sex (*F*_(1,26)_ = 0.962, *p* = 0.335) or nMK-801(p11–15) treatment interaction (*F*_(1,26)_ = 2.084, *p* = 0.159). **(C)** In the sociability test, there was a significant effect of nMK-801 (p11–15) treatment (*F*_(1,26)_ = 9.539, *p* = 0.004), but not effect of sex (*F*_(1,26)_ = 0.998, *p* = 0.324) or sex*treatment interaction (*F*_(1,26)_ = 4.103, *p* = 0.050). **(D)** In the social memory test here was a significant effect of treatment (*F*_(1,26)_ = 22.061, *p* < 0.001), but not an effect of sex (*F*_(1,26)_ = 0.831, *p* = 0.370) or sex*treatment interaction (*F*_(1,26)_ = 4.073, *p* = 0.052). ***p < 0.001.

In the sociability test, saline (p7–14)-treated female and male mice spent significantly more time exploring the stranger mouse than the empty box. However, both female and male nMK-801 (p7–14)-treated mice did not exhibit preference to the stranger mouse compared to the empty box, leading to a significant decrease in the discrimination indices [Figure 3A; significant effect of nMK-801 (p7–14) treatment (*F*_(1,26)_ = 15.264, *p* = 0.001), but not effect of sex (*F*_(1,26)_ = 3.809, *p* = 0.061) or sex*treatment interaction (*F*_(1,26)_ = 0.013, *p* = 0.909)]. In the social memory test, both saline (p7–14)-treated and nMK-801-treated mice interacted mostly with the newly introduced stranger mouse than the familiar one used [Figure 3B; significant effect of sex*treatment interaction (*F*_(1,26)_ = 10.303, *p* = 0.003), but no effect of sex (*F*_(1,26)_ = 0.962, *p* = 0.335) or nMK-801(p11–15) treatment interaction (*F*_(1,26)_ = 2.084, *p* = 0.159)].

There were no differences in the discrimination index of the sociability test between saline(p11–15)-treated and nMK-801(p11–15)-treated mice [Figure 3C; significant effect of nMK-801(p11–15) treatment (*F*_(1,26)_ = 9.539, *p* = 0.004), but not effect of sex (*F*_(1,30)_ = 0.998, *p* = 0.324) or sex*treatment interaction (*F*_(1,26)_ = 4.103, *p* = 0.050)]. However, there was a significant reduction in the discrimination indices of the social memory test between the saline(p11–15)-treated and nMK-801(p11–15)-treated female and male mice (Figure 3D) significant effect of treatment (*F*_(1,26)_ = 22.061, *p* < 0.001), but not an effect of sex (*F*_(1,26)_ = 0.831, *p* = 0.370) or sex*treatment interaction (*F*_(1,26)_ = 4.073, *p* = 0.052).

### Contextual Fear Conditioning

**Figure 4:**
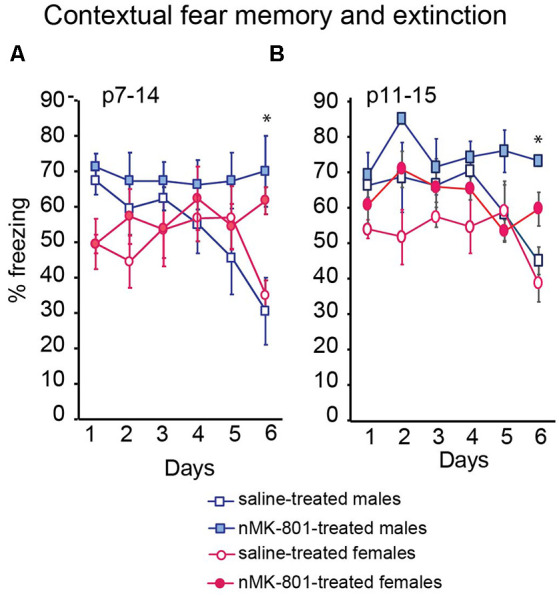
Effects of neonatal treatment with MK-801 at p7–14 **(A)** (*n* = 15) and p11–15 **(B)** (*n* = 15) compared to the respective saline-treated mice (*n* = 13 for each saline-treated group) on contextual fear memory and extinction. Open bars represent saline-treated mice, while colored bars represent the nMK-801-treated mice. **(A)** Repeated measures ANOVA analysis revealed day*treatment interaction (*F*_(1,26)_ = 7.552, *p* = 0.014). Per-day two-way ANOVA analyses showed that there was an effect of sex on day 1, no other effects on day 2–5 and a treatment effect on day 6 (*F*_(1,26)_ = 18.412, *p* = 0.001), but not an effect of sex or sex*treatment interaction (p7–14 model). **(B)** Repeated measures ANOVA analysis revealed day*treatment interaction (*F*_(1,26)_ = 6.552, *p* = 0.016). Per-day two-way ANOVA analyses showed that there was an effect of sex on day1, no other effects on day 2–5 and a treatment effect on day 6 (*F*_(1,26)_ = 15.334, *p* = 0.001), but not an effect of sex or sex*treatment interaction (p11–15 model). *p < 0.05.

The freezing levels of the saline (p7–14)-treated and nMK-801 (p7–14)-treated mice were not significantly different on day 1 following training. Saline (p7–14)-treated mice exhibited a reduction in freezing levels on day 6, compared to day 1, following training, indicating successful fear memory extinction. However, nMK-801 (p7–14)-treated mice exhibited significantly increased freezing levels on day 6, compared to saline-treated mice, indicating a reduction in fear memory extinction [Figure 4A; Repeated measures ANOVA analysis revealed day*treatment interaction (*F*_(1,26)_ = 7.552, *p* = 0.014). Per-day two-way ANOVA analyses showed that there was an effect of sex on day 1, no other effects on day 2–5, and a treatment effect on day 6 (*F*_(1,26)_ = 18.412, *p* = 0.001), but not an effect of sex or sex*treatment interaction)]. Similar results were observed in the nMK-801 (p11–15) female and male mice (Figure 4B) when compared to their controls [Repeated measures ANOVA analysis revealed day*treatment interaction (*F*_(1,26)_ = 6.552, *p* = 0.016). Per-day two-way ANOVA analyses showed that there was an effect of sex on day 1, no other effects on day 2–5, and a treatment effect on day 6 (*F*_(1,26)_ = 15.334, *p* = 0.001), but not an effect of sex or sex*treatment interaction)].

Object exploration was similar between the saline and nMK-801 treated mice, both p7–14 and p11–15 (Supplementary Figure 2). Furthermore, our results are not affected by differences in locomotor activity or anxiety between saline-treated and nMK-80-treated mice, both p7–14 and p11–15 (Supplementary Figure 3). Given the absence of differences on the effects of nMK-801 treatment between female and male mice, we continued our analysis without sex as a different factor.

### NMDA Receptor Subunits and Synaptic Proteins Status

**Figure 5:**
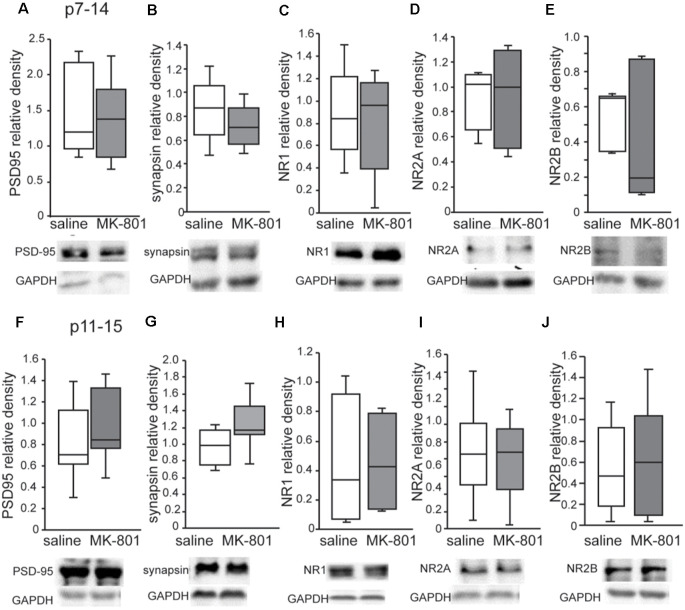
Expression of PSD-95, synapsin, NR1, NR2A, and NR2B in the PFC of nMK-801(p7–14; *n* = 6) and nMK-801 (p11–15; *n* = 6) compared to their respective saline-treated controls (*n* = 6 for each of the control group). **(A)** No differences are identified in the PSD95 relative density in the PFC of nMK-801 (p7–14) compared to saline-treated (*t*-test, *p* = 0.8). **(B)** No differences are identified in the synapsin relative density in the PFC of nMK-801 (p7–14) compared to saline-treated (*t*-test, *p* = 0.3). **(C)** No differences are identified in the NR1 relative density in the PFC of nMK-801 (p7–14) compared to saline-treated (*t*-test, *p* = 0.8). **(D)** No differences are identified in the NR2A relative density in the PFC of nMK-801 (p7–14) compared to saline-treated (*t*-test, *p* = 0.35). **(E)** No differences are identified in the NR2B relative density in the PFC of nMK-801 (p7–14) compared to saline-treated (*t*-test, *p* = 0.35). **(F)** No differences are identified in the PSD95 relative density in the PFC of nMK-801 (p11–15) compared to saline-treated (*t*-test, *p* = 0.4).** (G)** No differences are identified in the synapsin relative density in the PFC of nMK-801 (p11–15) compared to saline-treated (*t*-test, *p* = 0.15). **(H)** No differences are identified in the NR1 relative density in the PFC of nMK-801 (p11–15) compared to saline-treated (*t*-test, *p* = 0.9). **(I)** No differences are identified in the NR2A relative density in the PFC of nMK-801 (p11–15) compared to saline-treated (*t*-test, *p* = 0.8). **(J)** No differences are identified in the NR2B relative density in the PFC of nMK-801 (p11–15) compared to saline-treated (*t*-test, *p* = 0.9).

**Figure 6:**
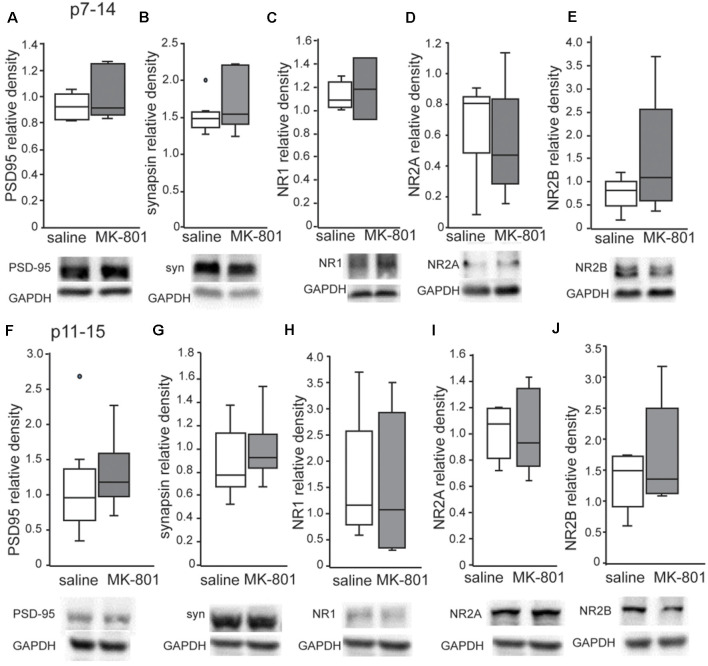
Expression of PSD-95, synapsin, NR1, NR2A and NR2B in the HPC of nMK-801 (p7–14; *n* = 6) and nMK-801 (p11–15; *n* = 6) compared to saline-treated controls (*n* = 6 + 6, 12 total). **(A)** No differences are identified in the PSD95 relative density in the HPC of nMK-801 (p7–14) compared to saline-treated (*t*-test, *p* = 0.4). **(B)** No differences are identified in the synapsin relative density in the HPC of nMK-801 (p7–14) compared to saline-treated (*t*-test, *p* = 0.4). **(C)** No differences are identified in the NR1 relative density in the HPC of nMK-801 (p7–14) compared to saline-treated (*t*-test, *p* = 0.6). **(D)** No differences are identified in the NR2A relative density in the HPC of nMK-801 (p7–14) compared to saline-treated (*t*-test, *p* = 0.5). **(E)** No differences are identified in the NR2B relative density in the HPC of nMK-801 (p7–14) compared to saline-treated (*t*-test, *p* = 0.2). **(F)** No differences are identified in the PSD95 relative density in the HPC of nMK-801 (p11–15) compared to saline-treated (*t*-test, *p* = 0.7). **(G)** No differences are identified in the synapsin relative density in the HPC of nMK-801(p11–15) compared to saline-treated (*t*-test, *p* = 0.3). **(H)** No differences are identified in the NR1 relative density in the HPC of nMK-801 (p11–15) compared to saline-treated (*t*-test, *p* = 0.75). **(I)** No differences are identified in the NR2A relative density in the HPC of nMK-801 (p11–15) compared to saline-treated (*t*-test, *p* = 0.9). **(J)** No differences are identified in the NR2B relative density in the HPC of nMK-801 (p11–15) compared to saline-treated (*t*-test, *p* = 0.2).

Western blot analysis revealed no changes of PSD95, synapsin, NR1, NR2A and NR2B levels in the PFC of both p7–14 (PSD95: *t*-test, *p* = 0.8, synapsin: *t*-test, *p* = 0.3, NR1: *t*-test, *p* = 0.8, NR2A: *t*-test, *p* = 0.35, NR2B: *t*-test, *p* = 0.35) and p11–15 (PSD95: *t*-test, *p* = 0.4, synapsin: *t*-test, *p* = 0.15, NR1: *t*-test, *p* = 0.9, NR2A: *t*-test, *p* = 0.8, NR2B: *t*-test, *p* = 0.9) nMK-801-treated mice when compared to their controls (Figure 5). In addition, we found no significant differences regarding PSD95, synapsin, NR1, NR2A, and NR2B levels in the HPC of both p7–14 (PSD95: *t*-test, *p* = 0.4, synapsin: *t*-test, *p* = 0.4, NR1: *t*-test, *p* = 0.6, NR2A: *t*-test, *p* = 0.5, NR2B: *t*-test, *p* = 0.2) and p11–15 (PSD95: *t*-test, *p* = 0.7, synapsin: *t*-test, *p* = 0.3, NR1: *t*-test, *p* = 0.75, NR2A: *t*-test, *p* = 0.9, NR2B: *t*-test, *p* = 0.2) nMK-801-treated mice when compared to their saline-treated mice (Figure 6).

### Parvalbumin (PV)

**Figure 7:**
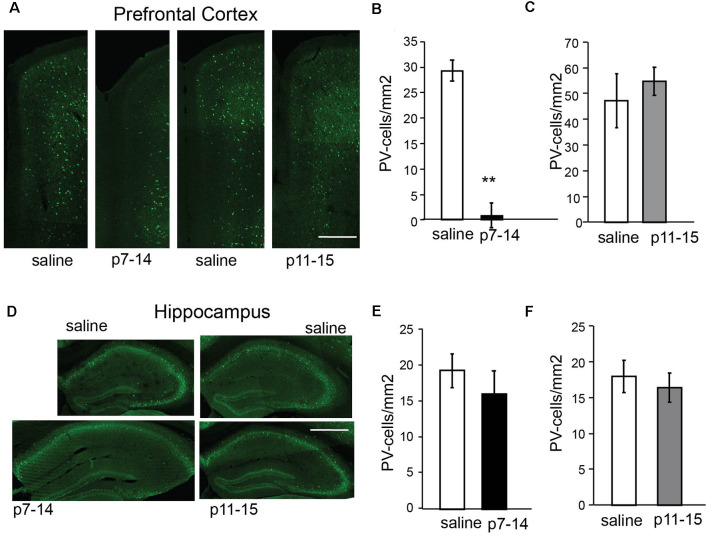
Number of PV-expressing cells in the PFC and HPC of nMK-801 (p7–14; *n* = 3) and nMK-801 (p11–15; *n* = 3) compared to saline-treated controls (*n* = 6 total, pooled from both p7–14 and p11–15 groups). **(A–C)** Representative images (scale bar: 0.5 mm) **(A)** and graphs showing that the number of PV-expressing cells is reduced in the PFC of nMK-801 (p7–14)-treated mice are reduced compared to their saline controls (*t*-test, *p* = 0.002) **(B)**, while the number of PV-expressing cells in the nMK-801 (p11–15)-treated mice is not affected compared to saline-treated controls (*t*-test, *p* = 0.3) **(C)**. **(D–F)** Representative images (scale bar: 1 mm)** (D)** and graphs showing that the number of PV-expressing cells is not different in the HPC of nMK-801 (p7–14)-treated mice compared to their saline controls (*t*-test, *p* = 0.5) **(E)**, or the HPC of nMK-801 (p11–15)-treated mice compared to saline-treated controls (*t*-test, *p* = 0.2) **(F)**. **p < 0.01.

The number of PV+ cells in the PFC of nMK-801(p7–14)-treated mice was significantly reduced when compared to saline (p7–14)-treated mice (Figures 7A,B; PFC: *t*-test, *p* = 0.002, HPC: *t*-test, *p* = 0.5). In the HPC, no significant differences in the number of PV+ cells of nMK-801 (p7–14)-treated mice were identified when compared to their controls (Figures 7D,E). The nMK-801 (p11–15)-treated mice showed no significant differences in the number of PV+ cells in both the PFC (Figures 7A,C; PFC: *t*-test, *p* = 0.3, HPC: *t*-test, *p* = 0.2) and the HPC (Figures 7D,F) when compared to saline (p11–15)-treated mice.

### Spontaneous Activity Properties

**Figure 8:**
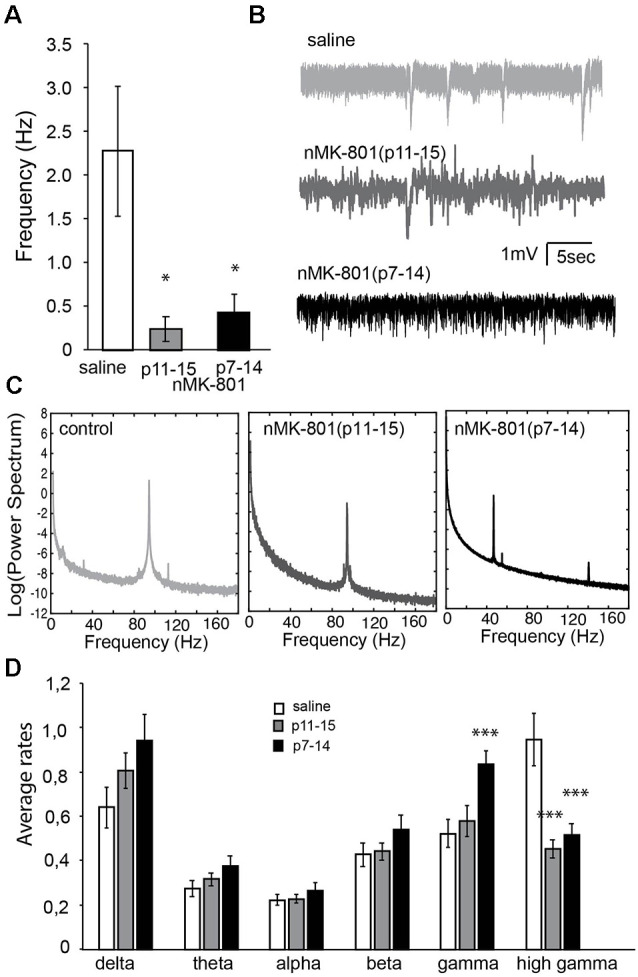
Properties of spontaneous activity in the PFC of nMK-801(p7–14; *n* = 6 animals, 12 slices) and nMK-801(p11–15; *n* = 6 animals, 12 slices) compared to saline-treated controls (*n* = 8 animals, 21 slices, pooled from both p7–14 and p11–15 groups). **(A)** Graph showing the frequency of up states is significantly different in nMK-801(p7–14) and nMK-801(p11–15)-treated mice compared to saline-treated (one-way ANOVA, *F*_(2,28)_ = 3.911 *p* = 0.032). *Post hoc* analysis (LSD test) shows a significant decrease in both the nMK-801(p7–14; *p* = 0.03) and nMK-801(p11–15)-treated mice (*p* = 0.03). **(B)** Representative traces of spontaneous activity in saline-treated, nMK-801(p7–14) and nMK-801(p11–15)-treated mice. **(C)** Representative traces of the power spectra from saline-treated, nMK-801(p7–14) and nMK-801(p11–15)-treated mice. **(D)** Graph showing the average rates of the different frequencies. There were no significant differences in the delta (*F*_(2,43)_ = 2.404, *p* = 0.108), theta (*F*_(2,43)_ = 1.596, *p* = 0.218), alpha (*F*_(2,43)_ = 0.380, *p* = 0.686), and beta (*F*_(2,43)_ = 1.022, *p* = 0.368) frequencies between nMK-801-treated and saline-treated mice. There was a significant effect of treatment in the gamma (*F*_(2,43)_ = 6.351, *p* = 0.004) and high gamma (*F*_(2,43)_ = 12.934, *p* = 0.0001) frequencies between nMK-801-treated and saline-treated mice. Specifically, in the gamma frequencies nMK-801(p7–14)-treated were significantly increased compared to saline-treated controls (LSD-test, *p* = 0.004) but not the nMK-801(p11–15)-treated mice (LSD test, *p* = 0.837). The high gamma frequencies, both nMK-801(p7–14) and nMK-801(p11–15)-treated mice were significantly reduced, compared to saline-treated mice (LSD-test, *p* = 0.0001 for both nMK-801-treated groups). ***p < 0.001; *p < 0.05.

Because we identified changes in the PV+-expressing cells in the PFC, we wanted to further investigate PFC function by studying the up states and neuronal synchronization in acute PFC brain slices. Incubation of PFC brain slices in a modified aCSF resulted in the generation of up states, as observed in previous studies (McCormick et al., 2003). The frequency of up states in nMK-801 mice (both p7–14 and p11–15) were significantly reduced, compared to saline-treated mice (Figures 8A,B; one-way ANOVA, *F*_(2,28)_ = 3.911 *p* = 0.032). *Post hoc* analysis (LSD test) shows a significant decrease in both the nMK-801(p7–14; *p* = 0.03) and nMK-801(p11–15)-treated mice (*p* = 0.03).

We further investigated the neuronal synchronization properties of spontaneous activity revealing differential effects in the various rhythms of synchronization. Power-spectra analysis (Figures 8C,D) shows that nMK-801 treatment, either p7–14 or p11–15, did not affect the delta (1–4 Hz), theta (4–7 Hz), alpha (8–12 Hz) and beta (13–30 Hz) rhythms in the PFC brain slices (no significant differences in the delta (*F*_(2,43)_ = 2.404, *p* = 0.108), theta (*F*_(2,43)_ = 1.596, *p* = 0.218), alpha (*F*_(2,43)_ = 0.380, *p* = 0.686), and beta (*F*_(2,43)_ = 1.022, *p* = 0.368) frequencies). However, both gamma (30–80 Hz) and high gamma (80–150 Hz) rhythms were significantly affected in a differential manner [significant effect of treatment in the gamma (*F*_(2,43)_ = 6.351, *p* = 0.004) and high gamma (*F*_(2,43)_ = 12.934, *p* = 0.0001)]. Especially, nMK-801(p7–14)-treated mice showed increased gamma activity compared to saline-treated (LSD-test, *p* = 0.004) and nMK-801(p11–15)-treated mice (LSD test, *p* = 0.837). On the other hand, both nMK-801(p7–14) and nMK-801(p11–15)-treated mice showed significantly reduced high gamma activity when compared to saline-treated mice (Figure 8D; LSD-test, *p* = 0.0001 for both nMK-801-treated groups).

## Discussion

Our work shows that neonatal treatment with MK-801 has differential effects on prefrontal cortical and hippocampal function, based on the temporal window of the treatment. Specifically, we find that nMK-801 treatment during p7–14 affects performance in all behavioral tasks examined, namely the novel object, the object-to-place, and the TOR tasks, as well as the sociability test and the contextual fear extinction. On the other hand, MK-801 treatment during p11–15 only affects performance in the TOR, the social memory, and the contextual fear extinction. These changes do not depend on alterations in NMDA receptor subunits expression or in synaptic protein expression. The earlier and long-term treatment [nMK-801(p7–14)] affected significantly the number of PV-expressing cells in the PFC, the frequency of up states, and also had larger effects on the gamma activity. On the other hand, the nMK-801(p11–15) treatment did affect the frequency of up states and the reduction of high gamma activity in the PFC.

### Sex Differences in SZ Animal Models

Most studies that include behavioral experiments in animal models of SZ have used mainly male rodents, especially rats. Thus, there is a lack of results on female rodents as well as on direct comparison between female and male subjects. Here, we discuss some studies that have included both male and female rodents in order to better understand the sex factor in these models.

The neurodevelopmental MAM16 mouse model of SZ revealed sex differences in PFC-dependent cognitive function, PFC proteome, and PV expression in the PFC and the HPC (Chalkiadaki et al., 2019). Moreover, prenatal PolyI: C treatment led to PPI sex-specific deficits (O’Leary et al., 2014; Ratnayake et al., 2014), to male-specific impairments in fear conditioning (Vorhees et al., 2012), in set-shifting task (Zhang et al., 2012), and in recognition memory test (Howland et al., 2012) as well as to female-specific dysfunction in spatial memory (O’Leary et al., 2014). Maternal immune activation also identified sex-specific deficits in PFC-dependent tasks (Pletnikov et al., 2008; Nelson et al., 2010; O’Leary et al., 2014; Hill, 2016). In addition, bacterial toxin lipopolysaccharide prenatal exposure was related to PPI and recognition memory impairments in a sex-specific manner (Wischhof et al., 2015). In the DISC1 genetic model of SZ, sex-dependent behavioral alterations were found in social interaction, hyperactivity, and in spatial memory (Pletnikov et al., 2008; Ayhan et al., 2011), and DISC1 male mutant mice were characterized by impaired synaptic activity in cortical pyramidal cells (Holley et al., 2013).

On the other hand, neonatal phencyclidine (PCP) administration revealed no sex differences on the increased locomotor activity, working memory deficits, and reduced social interaction behaviors (Hodes and Epperson, 2019). Double dose of MK-801 on p7 led to a decrease in hippocampal subiculum volume and in NR1 expression in both sexes during adulthood (Harris et al., 2003). MK-801 administration on p6 caused activation of caspase-3 in inferior colliculus 8 h later in male and female rats, while MK-801 administration on p6, p8, and p10 did not affect PPI performance on p28 or p56 of both sexes (Lyall et al., 2009). In addition, MK-801 treatment on p7 of Thy-1-YFP line H transgenic mice led to decreased layer V pyramidal neurons in the PFC and in decreased GABAergic transmission in the PFC on p82 in both sexes (Coleman et al., 2009). Thus, it is evident that postnatal treatment with MK-801 does not result in a sex-dependent phenotype in adulthood.

From the above discussion, it seems that sex differences emerge in several studies with prenatal manipulations while postnatal manipulations do not have a similar effect. It is possible that the absence of sex differences we observe in our behavioral experiments and other studies is due to the specific time period we perform the MK-801 treatment when the levels of estradiol between females and males are probably not significantly different (Bell, 2018). Estradiol could enhance the MK-801 affinity which might explain the higher vulnerability of females during adulthood (Hur et al., 1999).

### NMDA Receptors in MK-801 Treated Animal Models

MK-801 or dizocilpine is a non-competitive antagonist of NMDA receptors. Acute administration of MK-801 in adult rodents leads to SZ-like symptoms, such as increased locomotor activity and reduced PPI (Zhou et al., 2018). Evidence shows that the NR2B subunit of NMDA receptors, in particular, is correlated with cognitive impairments in SZ (Gilmour et al., 2012; Dauvermann et al., 2017). Specifically, a study showed that NR2B downregulation caused *disc1* (disrupted-in-schizophrenia 1) downregulation and reduced PPI performance (Zhou et al., 2018). On the other hand, the NR2A subunit is a faster target of MK-801, compared to the other NMDA receptor subunits (Gielen et al., 2009). NR2A subunits are highly distributed in GABAergic interneurons (Kinney et al., 2006; Xi et al., 2009). Interestingly, when MK-801 treatment is applied during early postnatal days for limited time periods, NMDA subunits levels are affected. Specifically, MK-801 administration three times on postnatal day 7 caused alteration in NR1 expression in the HPC, while MK-801 treatment during p6–21 led to long-term increase of NR1 in the cortex (Harris et al., 2003; Baier et al., 2009). Thus, it can be assumed that blockade of NMDA receptors during specific temporal windows of the postnatal period, can disturb the balance of excitatory-inhibitory signals affecting the neuronal survival and causing behavioral defects during adulthood, because of the persistent defects in NMDA receptor subunits expression. However, in this study, we did not identify any changes in the expression levels of either NMDA receptor subunits or synaptic proteins in the adult PFC and HPC, following NMDA blockade during p7–14 or p11–15. However, it is likely that subtler changes in NMDA receptor subunits have occurred, which cannot be measured by whole-tissue Western blot analysis.

### Schizophrenia and Network Synchronization

Spontaneous activity in cortical brain slices is characterized by up states, which are similar to awakeness, when information processing occurs and down states representative to sleep and anesthesia waves (Castro-Alamancos and Favero, 2015). Recent memory reactivation takes place during up states of slow oscillations and contributes to memory consolidation (Tatsuno et al., 2020). Up states have also been suggested to be an *in vitro* model of persistent activity, the cellular correlates of working memory (McCormick et al., 2003). An increase of beta/gamma power is observed during up states (Rebollo et al., 2018), which could be mediated by inhibitory interneuron activation (Neske and Connors, 2016).

NMDA receptors are implicated in the generation of up states and NMDA receptor blockade diminishes the frequency of up states in brain slices (Castro-Alamancos and Favero, 2015). Furthermore, blocking NMDA receptors *in vivo* results in an increase of gamma oscillations in the cortex while *in vitro* reduction in gamma activity is found following NMDA receptors blockade (Hakami et al., 2009; Olszewski et al., 2013). In addition, it has been shown that NMDA receptors blockade is linked with couple gamma independent oscillations *in vitro* and decreased delta rhythms (Kargieman et al., 2007; Anver et al., 2011). Interestingly, transgenic mice without NMDA receptors in PV-expressing interneurons are characterized by increased gamma oscillations and behavioral impairments (Carlén et al., 2012).

In general, abnormalities in neuronal oscillations are positively correlated with many neuropsychiatric disorders (Herrmann and Demiralp, 2005). Specifically, increased delta oscillations (Narayanan et al., 2015) and alterations in gamma rhythms (Herrmann and Demiralp, 2005; Uhlhaas and Singer, 2015) are observed in humans with SZ. Animal models of SZ have shown increased beta and gamma power (Roopun et al., 2008; Hakami et al., 2009; Olszewski et al., 2013; Molina et al., 2014). Additionally, the generation of up states is significantly altered in the MAM model of schizophrenia (Moore et al., 2006).

Our study shows reduced up state generation and differential regulation of gamma and high gamma activity in PFC brain slices. Therefore, it is possible that adaptations in up states and high gamma activity affect the function of the PFC, as indicated in both nMK-801 mouse models, while further adaptations in gamma activity and reduction in PV-expressing cells in the PFC behaviorally affect the function of both the PFC and the HPC, as shown in the nMK-801(p7–14) model.

### Differential Critical Periods for PFC and HPC Function?

Particular brain developmental processes occur within defined, narrow temporal windows suggesting the presence of critical periods for brain development. The critical period is “a strict time window during which experience provides information that is essential for normal development and permanently alters performance” (Hensch, 2005), or it is “a time window for great change and plasticity” (Meredith, 2015). Until now, there is no direct correlation between specific time-restricted critical periods and cognitive functions that depend on the PFC and hippocampus, the two main brain regions affected in SZ. In this study, we find differential cognitive deficits during adulthood depending on the specific temporal window of the MK-801 treatment. Disturbance of brain development during the early postnatal period (p7–11), affects both the prefrontal cortical and the hippocampal function during adulthood. However, MK-801 administration during p11–15 affects only the prefrontal cortical function during adulthood. Interestingly, when MK-801 treatment starts on p7 and ends on p14, the cognitive defects are stronger depicting the crucial role of HPC and PFC interaction for the higher cognitive function performance. These data suggest the possibility of the existence of different but also overlapping, critical periods for PFC and HPC development. However, our data on the number of PV-expressing cells do not directly support the above possibility, indicating that more subtle and complex neuronal relationships exist.

### Study Limitations

The conclusions of our study are subject to some limitations in our experimental design. First, our experimental design used a single daily MK-801 dose, instead of the double daily dosage used in other studies. Initial experiments which did use a double daily dose of MK-801(p11–15) in male mice suggested no difference between the single and double daily dose regimens on object recognition performance (Supplementary Figure 1). It is possible, however, that the double daily dose might have resulted in stronger neurobiological effects. Second, the duration of nMK-801 treatment was different between the two nMK-801 treatment groups. Specifically, in one group MK-801 was administered from p11 to p15 (i.e., 5 days) while in the other group MK-801 was administered from p7 to 15 (i.e., 8 days). It is possible that the treatment duration might have a larger contribution to the differences we see, compared to the different temporal periods. However, it seems that treatment duration does not have such a large effect in other studies. For example, the p1–22 MK-801 treatment (Facchinetti et al., 1993) had the same effects on locomotor activity in adulthood as the p3 MK-801 treatment (Beninger et al., 2002). Moreover, p7 MK-801 administration had the same effects on NR1 expression as the MK-801 treatment during p6–21 (Harris et al., 2003; Baier et al., 2009). Third, our behavioral results could be confounded by increased anxiety in nMK-801 treated mice. While we did not conduct an extensive analysis on anxiety, we did not find a significant difference in the thigmotaxis index in the open-field test conducted during habituation before the object recognition tests (Supplementary Figure 3). Fourth, our study design did not allow for testing in the adolescent period which is sensitive to the emergence of SZ symptoms. Future studies could address this very important issue. Finally, there is no clear mechanism by which nMK-801 treatment leads to behavioral impairments during adulthood. Understanding early-life brain development is critical towards a better knowledge of SZ etiology.

## Data Availability Statement

The raw data supporting the conclusions of this article will be made available by the authors, without undue reservation.

## Ethics Statement

The animal study was reviewed and approved by Protocol Evaluation Committee-Dept of Biology-University of Crete.

## Author Contributions

KS designed the study. MP, KD, CS, MV, and AG performed experiments. MP and KD analyzed data. VS and KS compiled the figures. MP, KD, VS, and KS wrote and edited the article. All authors contributed to the article and approved the submitted version.

## Conflict of Interest

The authors declare that the research was conducted in the absence of any commercial or financial relationships that could be construed as a potential conflict of interest.
