# Supplementary material for: Effect of Neonatal Treatment With the NMDA Receptor Antagonist, MK-801, During Different Temporal Windows of Postnatal Period in Adult Prefrontal Cortical and Hippocampal Function
Source: Front Behav Neurosci. 2021 Jun 11;15:689193. doi: 10.3389/fnbeh.2021.689193 (PMC8230549; doi:10.3389/fnbeh.2021.689193)
Supplement: Supplementary file 1 [file Data_Sheet_1.PDF]

Supplementary material

Methods

Single dose versus double dose of MK-801 treatment

In order to examine the dose-dependent effect of neonatal MK-801 treatment initially we conducted the MK-801 treatment of 0,1mg/kg twice a day in male mice during p11-15 and then we performed the object recognition tasks.

Locomotor activity

On p98 mice were habituated for 15 minutes in the open-field. Their locomotor activity was measured using JWatcher (https://www.jwatcher.ucla.edu/) estimating the moving or not moving status every 5 seconds for 15 minutes. The percentage of moving was calculated as: [(times found moving *100) / (times found moving+ times found not moving)].

Anxiety

To determine the anxiety status of the different groups of mice in the open-field, we measured the thigmotaxis index. For this, we analyzed the first 5 minutes of the first habituation trial that took place on p98 for the location of each mouse by JWatcher. The thigmotaxis index was calculated as: [(time spent in wall area + time spent in edges area) / (time spent in periphery area + time spent in center area)].

Exploration index

The test phases of Novel object, object to place and temporal order object recognition task were analyzed by JWatcher. The exploration index was measured as: (total time of exploration / time spent in the openfield).

Western blot

After antibody visualization on western blot membranes, the bands of interest were isolated and analyzed based on protein ladder bands. On supplementary figure 4 are represented raw data of membranes blotted with the antibodies of interest.

Results

Single vs double-dose study

Considering the different effects of dose frequency, we proceeded to a pilot experiment on p11-p15 treated males. We performed the object recognition tasks in Saline(p11-p15) and MK-801(p11-p15) single dosed and double doses -treated males during adulthood. The performance comparison between single dosed and double dosed was the same in all the three behavioral tests and depicted the results we observed in this paper (Supplementary figure 1).

Locomotor activity and anxiety in MK-801 treated groups

As locomotor activity and emotional status affect mice performance in behavioral experiments, the first day of habituation before the behavioral tests was analyzed for locomotor activity and thigmotaxis index. As represented in Supplementary figure 2A and 2B, nMK-801(p7-p14) treated mice had no statistically significant differences in locomotor activity and thigmotaxis when compared to Saline(p7-p14)-treated mice in both females and males. The same results were exhibited when Saline(p11-p15)-treated mice were compared to nMK(p11-p15)-treated mice (Supplementary figures 2C & 2D). These results suggest that the behavioral defects we observe in nMK-801-treated mice during the behavioral tasks are not related to other parameters than the MK-801 treatment affects.

Exploration during the object recognition tasks

Furthermore, the exploration index in each object recognition task was calculated to exclude the possibility of biased results. As it was expected there were no statistical significant differences between Saline(p7-p14)-treated and nMK-801(p7-p14)-treated mice both females and males in all object recognition tasks (Supplementary figures 3A, 3B, 3C). The same results were conducted for Saline(p11-p15)-treated and nMK-801(p11-p15)-treated mice in both females and males (Supplementary figures 3D, 3E, 3F). Thus, there were no differences in exploration between the mice groups that could have affected the observed results in object recognition tests.

Supplementary figure 1

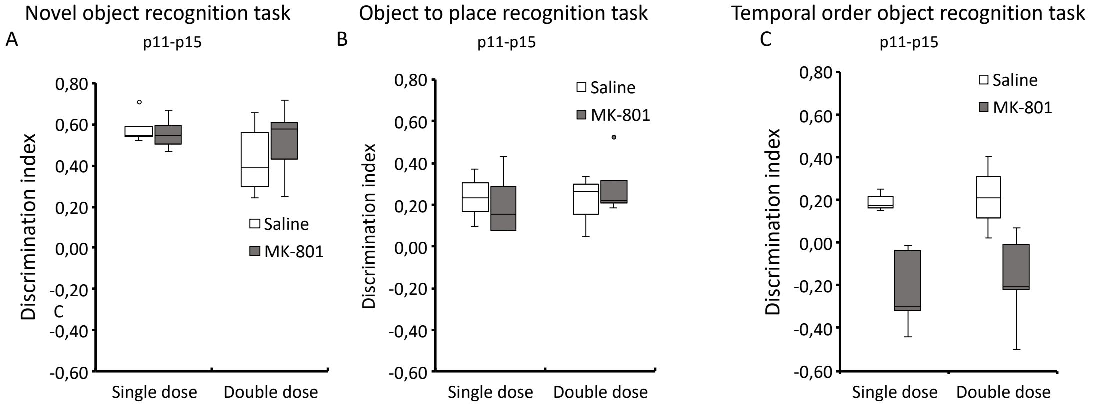

*Supplementary figure 1: Comparison of singled dosed and doubled dosed mice in Saline(p11-p15) and MK-801(p11-p15) treated males during adulthood. A) Discrimination index in novel object recognition task comparing the performance of Saline(p11-p15) and MK-801(p11-p15) treated male mice after using a single dose and a double dose protocol (two-way ANOVA group(F(1,14)=0.209), p=0.655); treatment(F(1,14)=1.960, p=0.183); group*treatment interaction(F(1,14)=0.672, p=0.426), B) Discrimination index in object to place recognition task comparing the performance of Saline(p11-p15) and MK-801(p11-p15) treated male mice after using a single dose and a double dose protocol (two-way ANOVA group(F(1,11)=0.073), p=0.792); treatment(F(1,11)=0.150, p=0.706); group*treatment interaction(F(1,11)=0.387, p=0.547), C) Discrimination index in temporal order object recognition task comparing the performance of Saline(p11-p15) and MK-801(p11-p15) treated male mice after using a single dose and a double dose protocol (two-way ANOVA group(F(1,12)=17.695), p=0.001); treatment(F(1,12)=0.141, p=0.714); group*treatment interaction(F(1,12)=0.028, p=0.870).*

Supplementary figure 2

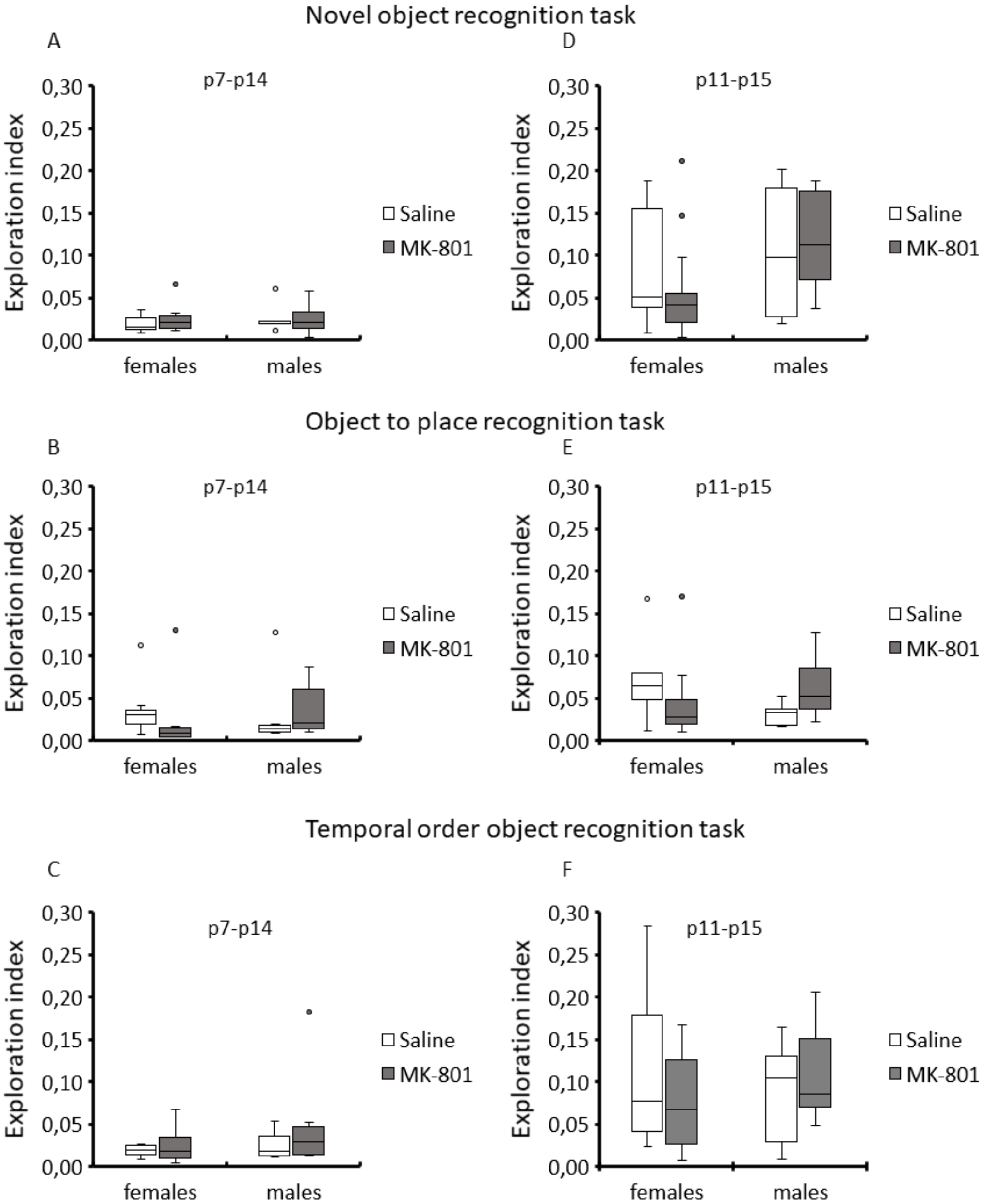

*Supplementary figure 2: A) Exploration index of Saline(p7-p14) treated and nMK-801(p7-p14) treated mice in both females and males during novel object recognition task (two-way ANOVA, sex(F(1,26)=0.097, p=0.758); group(F(1,26)=0.233, p=0.633); sex*group(F(1,26)=0.474, p=0.497), B) Exploration index of Saline(p7-p14) treated and nMK-801(p7-p14) treated mice in both females and males during object to place recognition task (two-way ANOVA, sex(F(1,26)=1.072, p=0.310); group(F(1,26)=1.151, p=0.293); sex*group(F(1,26)=0.229, p=0.636), C) Exploration*

*index of Saline(p7-p14) treated and nMK-801(p7-p14) treated mice in both females and males during temporal order object recognition task (two-way ANOVA, sex(F(1,26)=0.005, p=0.946); group(F(1,26)=0.019, p=0.892); sex*group(F(1,26)=0.196, p=0.662), D) Exploration index of Saline(p11-p15) treated and nMK-801(p11-p15) treated mice in both females and males during novel object recognition task (two-way ANOVA, sex(F(1,39)=4.218, p=0.047); group(F(1,39)=0.081, p=0.777); sex*group(F(1,39)=1.124, p=0.296), E) Exploration index of Saline(p11-p15) treated and nMK-801(p11-p15) treated mice in both females and males during object to place recognition task (two-way ANOVA, sex(F(1,35)=4.572, p=0.677); group(F(1,35)=0.086, p=0.771); sex*group(F(1,35)=4.572, p=0.040), F) Exploration index of Saline(p11-p15) treated and nMK-801(p11-p15) treated mice in both females and males during temporal order object recognition task (two-way ANOVA, sex(F(1,37)=0.068, p=0.796); group(F(1,37)=0.149, p=0.702); sex*group(F(1,37)=2.066, p=0.159).*

Supplementary figure 3

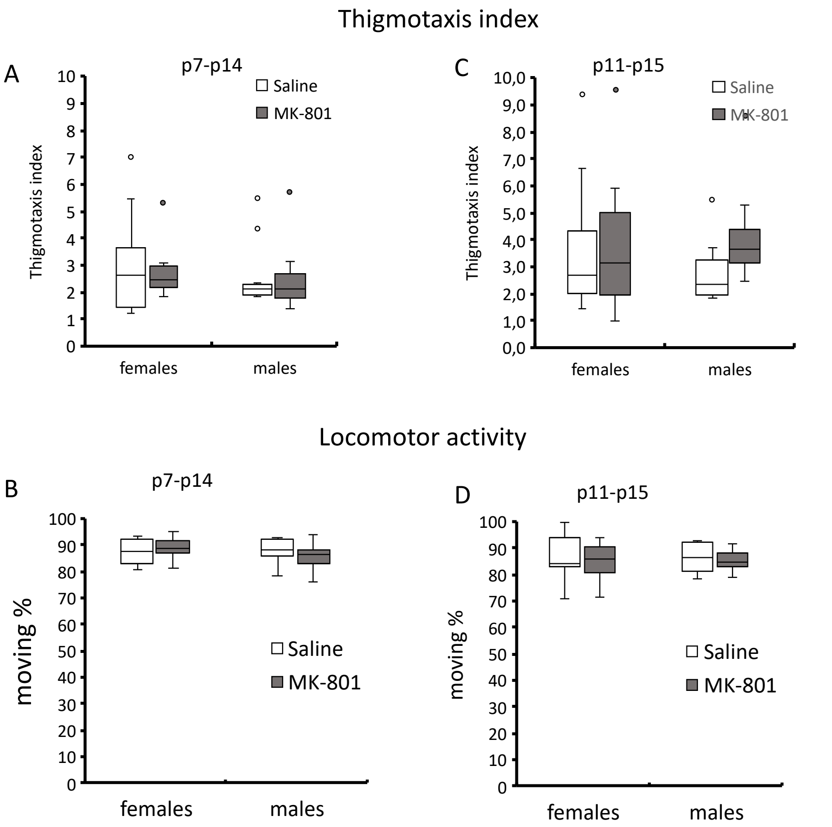

*Supplementary figure 3: A) Thigmotaxis index of nMK-801(p7-p14) compared to Saline(p7-p14) treated mice in both females and males on p98 (two-way ANOVA, sex (F(1,35)=1.217, p=0.277); group(F(1,35)=0.107, p=0.745; sex*group(F(1,35)=0.019, p=0.891), B) Locomotor activity of nMK-801(p7-p14) and Saline(p7-p14) treated mice in both females and males on p98 (two-way ANOVA, sex (F(1,35)=0.730, p=0.399); group(F(1,35)=0.168, p=0.684; sex*group(F(1,35)=1.213, p=0.278), C) Thigmotaxis index of nMK-801(p11-p15) compared to Saline(p11-p15) treated mice in both females and males on p98 (two-way ANOVA, sex (F(1,40)=0.308, p=0.582); group(F(1,40)=2,538 p=0.119); sex*group(F(1,40)=2.845, p=0.099), D) Locomotor activity of nMK-801(p11-p15) and Saline(p11-p15) treated mice in both females and males on p98 (two-way ANOVA, sex(F(1,40)=0.003, p=954); group(F(1,40)=0.747, p=0.393); sex*group(F(1,40)=0.015, p=0.902).*

Supplementary figure 4

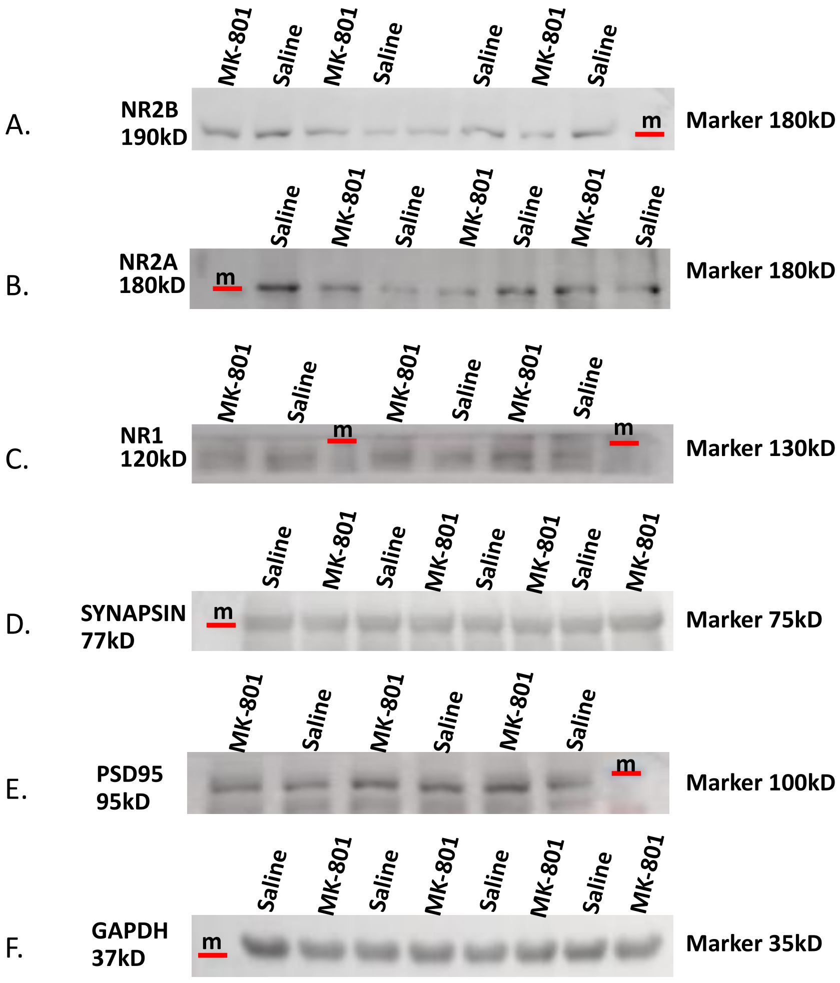

Supplementary figure 4: Western blot visualized membranes. A) Membrane blotted with NR2B primary antibody on the whole cell extracts of the HPC of the Saline(p11-p15) and MK-801(p11-p15) – treated females and males, B) Membrane blotted with NR2A primary antibody on the whole cell extracts of the HPC of the Saline(p7-p14) and MK-801(p7-p14) – treated females, C) Membrane blotted with NR1 primary antibody on the whole cell extracts of the HPC of the Saline(p7-p14) and MK-801(p7-p14) – treated females, D) Membrane blotted with SYNAPSIN primary antibody on the whole cell extracts of the HPC of the Saline(p11-p15) and MK-801(p11-p15) – treated females and males, E) Membrane blotted with PSD95 primary antibody on the whole cell extracts of the PFC of the Saline(p7-p14) and MK-801(p7-p14) – treated males, F) Membrane blotted with GAPDH primary antibody on the whole cell extracts of the HPC of the Saline(p11-p15) and MK-801(p11-p15) – treated females and males.
